# From Languishing Dyslexia to Thriving Dyslexia: Developing a New Conceptual Approach to Working with People with Dyslexia

**DOI:** 10.3389/fpsyg.2015.01976

**Published:** 2015-12-24

**Authors:** Chathurika S. Kannangara

**Affiliations:** Department of Psychology, University of BoltonBolton, UK

**Keywords:** dyslexia, positive psychology, strengths, thriving dyslexic, higher education, dyslexia Intervention

## Abstract

This is an account of personal narratives shared by several people with dyslexia. Most of these are presented in their original quotation format to provide personal accounts of the lives of people with dyslexia. In this paper the author shares her conversations with her participants. This paper provides an original conceptual model, which is currently been tested empirically. Dyslexia affects the learning process in areas as such reading, and spelling. Conversely abilities or strengths can be seen in other areas, such as developing coping strategies to manage and overcome challenges. This research aims to adapt positive psychology techniques to support individuals with dyslexia. To develop positive psychology interventions, individuals will be helped to discover their five signature strengths. The VIA (Values in Action) Strengths Survey has been hosted in a website which has been developed in the form of a dyslexia user friendly format, such as providing the ability for respondents to change fonts and font sizes, colors and a text to speech option. This paper introduces the theoretical model of ‘How to move from Languishing Dyslexia to Thriving Dyslexia.’

“I am known today to be a perfectionist, keen on documentation, stickler to the rules of language, be it grammar or spellings.”

“…and yet I was the same child benumbed by fear, standing up in class, trying to maintain any shred of dignity through the humiliating experience of being laughed at my attempt to read aloud. I had mispronounced the word ‘native’ and my well-kept secret was out in Grade 3.”

These are some of the quotes included in a narrative of ‘My Life with Dyslexia’ written by one of the lecturers I met in my Masters course. During an informal discussion with Dr. Shailaja Shastri while I was doing my MSc in Psychology, she explained the painful reality of her life growing up with dyslexia. This led me to reflect, not only about her personal struggle but made me wonder how she had gone from early struggles to become an impressive Psychology professor? She was the person who was now helping to build my confidence in exploring the field of Psychology. When I started my PhD in Bolton, UK, she was very happy to provide me with the narrative story of her ‘Life with Dyslexia’ as part of my PhD studies. What interested me more was how dyslexia has become an inter-generational challenge, since her only son was also diagnosed with dyslexia. Both mother and son have endured similar battles with their diagnoses. Her son, Sanjal Shastri, is also a young scholar currently studying for his Masters at the London School of Economics. When I asked him for his narrative story of having a ‘Life with Dyslexia,’ he identified his title of the narrative story to be, *‘I define my life not dyslexia’* and further he says,

“*I realized there was something wrong when I used to face difficulty in answering dictation tests. In school I had to face the constant remark from my teachers stating how bad my handwriting was.”*

“I was able to overcome all these challenges through practice.”

For me the most interesting statement he made in his narrative was,

“The key to overcoming dyslexia is by changing your mindset.”

Dweck (2012) introduces changing ‘Mindsets’ as an intervention for children to excel in education. Even though Sanjal Shastri’s primary studies are within economics, he embraces a psychological concept, i.e., ‘mindsets,’ the concept that he embraced to change the impact of dyslexia in his life was changing his ‘mindset’ thus building a bridge to the work of Dweck (2012).

Another scholarly woman from the field of Economics writes in her narrative

“ … nearing the end of fourth decade in my life. Still my childhood experiences can bring me to tears.”

“I seemed to stand out as a sore thumb, the misfit, an ugly (read ‘dumb’) duckling among elegant (read ‘intelligent’) swans.”

“….now Head of the Department (in a well known university, Economics department) no one would ever doubt my scholastic abilities….”

While working as a trainee counselor in India and Sri Lanka I frequently met students with dyslexia within the therapeutic centers. Dyslexia has correlations with a number of disadvantages and difficulties. Mental health classifications (such as the International Classification of Diseases and the Diagnostic and Statistical Manual of Mental Disorders, DSM V) and working definitions (Eg: definition by British Dyslexia Association) either provide a discrepancy criterion or disability-based criterion for diagnosis. However, a dyslexia diagnosis is very commonly associated with average or in many cases above average levels of intelligence (American Psychiatric Association, 2013; Bdadyslexia.org.uk, 2015). Dyslexia has become a diagnosis, which has seen its numbers rise rapidly in recent years in the UK (Michail, 2015). With numbers steadily increasing, I wondered whether there was any other way to consider, exactly ‘what is dyslexia?’ There are many life stories of highly successful individuals who have stated that they have battled against dyslexia, including famous entrepreneurs like Richard Branson. The journalist Malcolm Gladwell very interestingly answered the question I was wondering about, in his book, ‘David and Goliath’ (Gladwell, 2014). In this he referred to dyslexia as a desirable disorder. Amongst other individuals mentioned in the book he talks about who have turned their “disability” to their advantage, is the American lawyer David Boies. Gladwell’s term ‘desirable disorder’ has inspired my work on dyslexia. As a result of that, I have set up a website to get participants for my PhD research from all over the world. which I named as www.desirabledyslexia.com.

In the process of discovering more about dyslexic abilities (Alexander-Passe, 2010) and nature of their lives, I started to learn that there are many gifted abilities or compensatory strategies that people develop. The narrative stories I have collected and the stories and research written by entrepreneurs (Logan, 2009) have directed my PhD research toward the field of Positive Psychology, and a possible synergy between this and dyslexia. The first step was to attempt to identify Signature Strengths among individuals with Dyslexia using the Values in Actions (VIA) Inventory, developed by Professors Martin Seligman and Christopher Peterson (Peterson and Seligman, 2004; Niemiec, 2013). Professor Seligman and VIA Institute gave me permission to develop a user-friendly version of the VIA Strengths Inventory. This allows individuals with dyslexia to change font sizes, background colors, use a text to speech option etc., so the test is easier to complete. Additionally I have been inspired to develop a Positive Psychology based intervention for Dyslexia. This led me to move my work on dyslexia away from traditional disability based definitions. I realized that a possible *flourishing* model for dyslexia would direct as well as support dyslexics towards their personal exploration of strengths as well as utilizing their strengths to thrive in life.

While considering possible Positive Psychology Interventions (PPI) for dyslexia, it was important to focus on exiting remedies and techniques of support, which have widely been used for dyslexia. One among them is Positive Behavior Interventions (PBI) or Positive Behavior Intervention and Support (PBIS), is another approach in high demand, with applications are widely used in many educational environments. PBI emphasizes on positive techniques such as reinforcement, motivation etc., PBIs differs from PPIs, with the latter’s focus being augmenting functioning of the self, ‘what is able’ and not necessarily reparative ‘what is dis-able.’ Unlike PBIs are used for providing remedies for the problematic behaviors (Carr, 1999). According to Sin and Lyubomirsky (2009) PPIs aim to enhance wellbeing by increasing positive affect, cognition, and behaviors. PPI also works toward enabling individuals to achieve and function at their optimal self by developing hope, purpose, and mastery of life. The reparative nature of the PBI aims to address the symptomatic behaviors associated with disorders. The current study focuses on a PPI based approach for people with dyslexia that goes beyond addressing symptomatic behaviors associated with dyslexia. Thus making it one of its kind model that defocuses from a traditional reparative approach for addressing dyslexia and instead looks at concepts or scaffolding behaviors like signature strengths, grit, hope, and growth mindset, to enable dyslexics to achieve and work at their optimal self.

I have identified two broad, but distinct types of dyslexic personalities based on their approach toward understanding how dyslexia affects them and the strategies they have evolved to cope with it. The two distinct personalities among dyslexics are ‘Languishing Dyslexics’ and ‘Thriving Dyslexics.’ However, the proposed model provides a classification and also delineates steps to transition from a ‘languishing state to the ‘Thriving’ counterpart. Preliminary observations based on narratives of successful dyslexics and the existing literature on the condition, have led to the consolidation of characteristics peculiar to each of the two types of dyslexics, written by successful dyslexics. The literature about dyslexia has led to the identification of some characteristics of languishing dyslexics and thriving dyslexics.

My new work is similar to the work of Dweck (2012), mentioned above, who proposed an interesting model called ‘mindsets’ to categorize perceptions about one’s intelligence. Dweck (2012) has identified two types of mindsets; fixed and growth mindsets. Her model focuses on “…perceptions of and beliefs about one’s intelligence especially with regard to its inherent ability to improve with practice.”

I believe that my work is an extension of how these mindsets along with other (overt/covert) behaviors play out in dyslexics. I propose that the languishing dyslexic works (predominantly) from a fixed mindset, while a growth mindset guides a thriving dyslexic. A thriving dyslexic is a strong reflection of an individual with a growth mindset, who works around the condition of dyslexia, effectively problem solves and is inspired by the belief that they are not all about ‘what’s wrong,’ but also possess unique strengths to override the wrong. While a languishing dyslexic “passes the buck” and succumbs to the challenges posed by the condition.

The focus on one’s signature strengths is echoed in one of the guiding principles of positive psychology as described by Professor Martin Seligman and Dr Christopher Peterson (Peterson and Seligman, 2004; Seligman, 2011).

A languishing dyslexic is anxious about facing challenges and therefore either avoids them or engages in distracting behaviors when faced with a challenge, especially when they are given a task of reading and writing. They are burdened by obstacles, criticisms are ignored or take an emotional toll, and they indulge in self-deprecation and give up in the aftermath of failure.

In contrast to a languishing dyslexic, a thriving dyslexic predominantly displays characteristics such as positive acceptance toward challenges, embraces them, uses signature strengths when they come across obstacles, learns from criticisms, perseveres, withstands, and finds alternative approaches when they face failures.

My PhD research aims to test the above model (**Figure 1**), by introducing a PPI to change the languishing experience into a thriving experience for people with dyslexia. The change of perspective in a dyslexic is achieved by implementing PPIs to flourish. My research aims are to introduce this model for managing and working with dyslexia in a more positive independent way. This will deviate from the traditional disability model avoiding extreme dependency on diagnosis or the label of dyslexia and instead promoting ‘signature’ strengths. According to Professor Seligman signature strengths are unique to each of us, and if we build on them, they will lead us to thrive in life.

**FIGURE 1:**
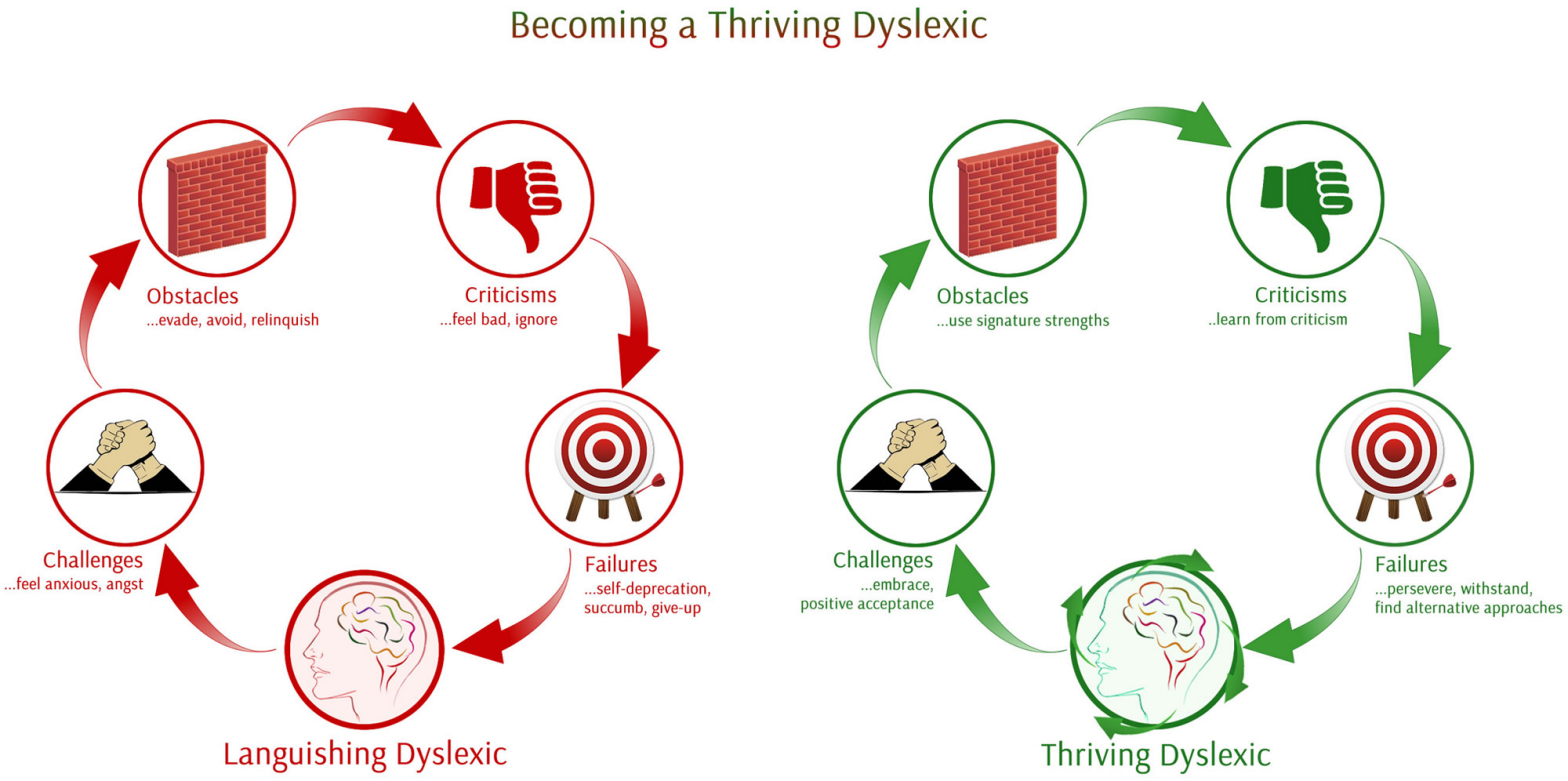
**Moving from languishing to thriving dyslexia**.

The proposed intervention aims at enabling people with dyslexia; to firstly discover their signature strengths and then will be trained in how to best use them in daily life. For instance, how to develop growth mindset to look beyond obstacles. Another focus area would be how to develop grit so as to persevere in academia. Thus a cluster of positive psychology techniques would contribute to making a significant change to the self-concept of a person with dyslexia. Also such an intervention hopes to tangentially make a significant change to others perception toward dyslexia. The model would thus serve as a customized route map to becoming a thriving dyslexic. According to existing literature and after careful consideration on existing techniques to help dyslexia, I believe that this is the first attempt to merge positive psychology with dyslexia in order to help individuals with dyslexia with a model that identifies two kinds of people with dyslexia.

In 2 years from now, I am hoping that this model will be fully tested and empirically strengthened to identify possibilities and probable implications in the field of education and career development for dyslexics.

One among a few successful dyslexic participants – who I consider a ‘thriving dyslexic’, wrote:

“I don’t crave for stability or security as I see most people do, I don’t cry over spilt milk, I don’t accept helplessness in people. I have learnt that the only entity you can count on at the end of the day is you not even your abilities. Because there will always be and most often is someone better than you, all you can do is be better than your previous attempt.”

“I consider success and failure as transient states I don’t get swayed by either. That does not mean I don’t have ambitions I don’t have desires, I do but I possess them and define them, they do not possess or define me.”

## Conflict of Interest Statement

The author declares that the research was conducted in the absence of any commercial or financial relationships that could be construed as a potential conflict of interest.
